# Ginger Powder-Based Pickering Emulsions: An Innovative Platform for Anticancer Drug Delivery

**DOI:** 10.3390/molecules30224349

**Published:** 2025-11-10

**Authors:** Patrizia Formoso, Domenico Mammolenti, Adele Chimento, Maria Carmela Pellegrino, Ida Daniela Perrotta, Francesca Romana Lupi, Domenico Gabriele, Vincenzo Pezzi

**Affiliations:** 1 Department of Pharmacy, Health and Nutritional Sciences, University of Calabria, 87036 Rende, CS, Italy; adele.chimento@unical.it (A.C.); marapellegrino97@gmail.com (M.C.P.); v.pezzi@unical.it (V.P.); 2 Department of Information, Modeling, Electronics and System Engineering, (D.I.M.E.S.), University of Calabria, Via P. Bucci, Cubo 39C, 87036 Rende, CS, Italy; domenico.mammolenti@unical.it (D.M.); francesca.lupi@unical.it (F.R.L.); 3 Centre for Microscopy and Microanalysis (CM2), Department of Biology Ecology and Earth Sciences, University of Calabria, 87036 Rende, CS, Italy; ida.perrotta@unical.it

**Keywords:** Pickering emulsion, ginger, natural emulsifier and stabilizer, doxorubicin loading and release, targeted drug delivery systems, controlled antineoplastic drug release

## Abstract

Biodegradable Pickering emulsions are attracting increased appeal owing to their promising and diversifying therapeutic applications. In this study, for the first time, a novel therapeutic Pickering emulsion stabilized with ginger powder (GA4) was formulated, characterized, and tested for doxorubicin (DOX) delivery. GA4_Pes physicochemical characterization by DLS (Dynamic Light Scattering), POM (Polarized Optical Microscopy), Cryo-SEM (Cryo-Scanning Electron Microscopy), TEM (Transmission Electron Microscopy), and rheology testing confirmed stability for at least one month, solid-like gel properties, and multiple morphology even at a low concentration of stabilizer. In addition, the morphological, dimensional, and rheological properties of some GA4_Pe loaded with DOX (GA4_Pe@DOX) were examined. These formulations were of the w/o/w type, stable for at least 28 days, and showed efficient doxorubicin internalization. A 24 h in vitro release assay displayed a sustained and pH-dependent release, with 30% and 50% chemotherapeutic released at pH 7.4 and 5.6, respectively. Furthermore, in vitro cell viability assessment performed using GA4_Pe showed no toxicity on immortalized 3T3 mouse embryonic fibroblasts but a small significant inhibitory effect on human breast cancer cell line MCF7. Interestingly, the GA4_Pe@DOX emulsion exerted a cytotoxic effect on MCF7 cells very similar to that of the free DOX solution with the same doses of DOX loaded in the same emulsion. Therefore, the total biocompatibility/biodegradability, good drug entrapment, and high stability, as well as the prolonged release and anti-tumor efficacy maintenance of the loaded drug, suggest a feasible application of ginger powder-based Pickering emulsions for topical delivery as a selective therapeutic platform in targeted formulations of antineoplastic drugs.

## 1. Introduction

In recent decades, the pharmaceutical industry has shown considerable interest in research on the formulation and development of controlled drug release systems based on Pickering technology [1,2,3], which makes use of solid nano- and micro-particles instead of synthetic surfactants to stabilize emulsions. These solid particles (named Pickering particles) induce higher long-term stability against emulsion coalescence because they are more strongly adsorbed at the interface of the dispersed phase droplets. Additionally, they are both more biosecure and environmentally friendly than conventional and molecular surfactants. The surfactant-free character and enhanced physical stability of Pickering emulsions (Pes) make them a challenging opportunity for topical formulations whit the aim of realizing biocompatible and sustainable transdermal drug delivery systems (BS_TDDSs) [4,5]. Therefore, the nature of solid Pickering particles is undoubtedly a critical factor that strongly affects the performance of biodegradable Pickering emulsions such as BS_TDDSs.

Many researchers have abandoned the use of synthetic and inorganic conventional solid Pickering particles, such as silica, carbon nanotubes, and titania [6], in favor of more biocompatible organic emulsifiers/stabilizers, such as starch, chitosan, and cellulose particles, lipids, proteins, and cyclodextrins, for targeted delivery of nutraceuticals in pharmaceutical applications [7,8], and recently also in food areas [9,10]. Lately, plant particles, which are naturally rich in antioxidants, were proposed as dual-functional Pickering particles [11] that act both as physical emulsion stabilizers and as interfacial reservoirs of bioactive compounds. Although studies of natural particle-based Pickering emulsions have been carried out widely, there is a limited literature available on their topical pharmaceutical applications [12,13,14]. Nevertheless, the use of natural plant particle-based Pickering emulsions may be a novel strategy for topical drug administration that could make possible the direct application of active molecules to the target site of action while minimizing systemic side effects. Natural food-grade emulsifiers are more environmentally and skin-friendly with respect to other Pickering particles, in addition to being non-carcinogenic; thus, their high biocompatibility, biodegradability, and excellent oxidation resistance are promising characteristics for the development of biologically proper Pickering emulsions as a drug delivery platform that can allow for controlled and delayed topical release of chemotherapeutic agents. However, to the best of our knowledge [15,16], until now, there have been no reports on the use of natural food grade particle-stabilized Pickering emulsions (FGPS_Pes) for skin application of natural anti-cancer drugs such as anthracyclines.

Doxorubicin (DOX) is a hydrophilic molecule that belongs to the anthracycline class of drugs. Although considered a natural anti-tumor antibiotic because it is isolated from Streptomyces peucetius var. caesius, DOX is associated with neutropenia and cardiotoxicity in clinical applications [17]. Doxorubicin (DOX) is a well-known anti-neoplastic drug with a good broad-spectrum antitumor activity [18]; although it is reported as the first-line chemotherapy drug in the treatment of leukemia, sarcoma, and breast cancer [19,20,21], it has relevant drawbacks, including severe and adverse side effects such as accumulation at non-tumor sites and poor selectivity.

A transdermal route of administration of DOX can certainly subdue its well-known cardiotoxicity, although its high hydrophilicity certainly does not promote skin permeation. Over the past forty years, considerable efforts have been made to identify appropriate nanoformulations, with permeation strategies that go beyond simply reducing the size to the nanoscale [22]. Thus, self-assembled nanomaterials, such as surfactant-based nanoemulsions [23,24], lipid-based nanocarriers (niosomes and liposomes) [25,26], and polymer-based nanoparticles [27], have been proposed as potential candidates for low-dose transdermal delivery of DOX, as they work as smart drug delivery systems. DOX delivery can be facilitated by selectively activating smart drug delivery platforms, which is only achievable if nanoformulations are able to respond to internal (pH, temperature, redox conditions, enzymatic activity, etc.) or external (mechanical pressure, ultrasound, magnetic fields, electric fields, and UV, Vis, or NIR light) stimuli [28]. Recently, new topical nanoformulations have been designed for the co-delivery of DOX and other drugs (both chemotherapeutic and non-chemotherapeutic) for a combination therapy aimed at improving therapeutic efficacy and overcoming systemic toxicity [29].

Some multifunctional therapeutic systems exploit synergistic chemo/photodynamic and immune/photodynamic effects to target superficial tumors via the transdermal route but do not always reduce toxic side effects such as skin irritation due to the phototoxicity of the photosensitizer [30,31]. Other multitherapeutic nanoformulations propose the cardioprotective action of antioxidants to reduce the DOX cardiotoxicity side effects [32,33,34].

We believe that the use of the FGPS_Pes may represent an innovative and promising approach when the primary objective is to design a less invasive but equally effective method of topical administration of antineoplastic drugs such as DOX. A therapeutic treatment based on naturally antioxidant-rich FGPS_Pes has the potential to reduce post-treatment debacles, improving the patient’s conditions and quality of life. To date, a few Pe-based doxorubicin delivery systems have been reported for cancer treatment, but their therapeutic application remains limited to transarterial chemoembolization (TACE) treatment by lipiodol-based Pes [35,36], in addition to the more recently reported intratumoral administration of ethiodized oil-based Pes [37].

Stimulated by the need to reduce the side effects resulting from DOX systemic distribution and to enhance its site-specific cytotoxicity in a targeted topical delivery system, the aim of this study was to assess the DOX delivery properties of FGPS_Pes prepared from ginger nanoparticles (GA4). Thus, various amounts of GA4 were used to stabilize ginger powder-based Pickering emulsions (GA4_Pes) loaded with doxorubicin. After physicochemical characterization over time, the impact of the GA4 concentration and water/oil ratio on the drug release properties of DOX-loaded GA4_Pes were investigated. The following properties of DOX-loaded and -unloaded GA4_Pes were studied up to one month after preparation: physical stability, droplet size distribution, and surface charge. Meanwhile, rheological measurements were performed to support the conclusive discussion of the drug delivery behavior of DOX loaded GA4_Pes. Lastly, the potential of this novel and eco-friendly GA4_Pe-based novel and ecofriendly system to load and release DOX while reducing toxic side effects was evaluated in vitro and compared with that of DOX solution.

## 2. Results and Discussion

### 2.1. GA4-Based Pickering Emulsion Formulation

The good bifunctional performance of ginger particles as Pickering emulsion stabilizers was highlighted in a previous report [11]. Ginger powder from conventional farming for a local trade chain, named GA4, can work very well as an interfacial reservoir of bioactive miscellaneous (proteins, polysaccharides, and antioxidants) without changing its stabilizing and emulsifying properties. The one-step emulsification process performed under a protective atmosphere by mixing the water, oil, and the particles at the same time allowed particles to acquire different contact angles during emulsification, inducing the possible formation of a Pickering double emulsion [3,38,39]. Certainly, the one-step strategy induced a change in the morphology of the GA4 powder, which originally contained particles of different sizes, surface structures, and shapes. The mixing intensity setup of one-step emulsification entailed fine particle fragmentation and good disentanglement of some large primary particles, leaving very few residues of particle aggregates larger than 2 μm, also in a GA4 aqueous dispersion (1%) if submitted to same one-step treatment [11]. DLS analysis showed that the processed GA4 powder presents a rather homogeneous and narrow particle distribution, exhibiting a PdI (polydispersity index) of 0.15 ± 0.01 and an average hydrodynamic size of 370 ± 20 nm (Table S1). According to contact angle and Zeta potential analysis (Table S1), GA4 particles showed a moderately amphiphilic character that favored the formation of very stable W/O/W emulsions over time [11]. In particular, in a storage stability study of GA4-based Pickering emulsion samples at different φ_w_ values for up to 3 months, a relevant stability to coalesce (no phase separation) was observed in the GA4_Pe sample at an oil-water ratio (*v*/*v*) of 1/1 prepared with 2% *w*/*w* of GA4 [11].

Therefore, in order to identify an optimized GA4_Pe formulation for DOX loading, in this study, three distinct GA4 concentrations (15, 20, and 40 mg/mL) were initially tested, and their influence was evaluated by tracking the physical stability and droplet size distribution of the resulting GA4_Pes over time.

### 2.2. GA4_Pes Characterization

**Figure 1:**
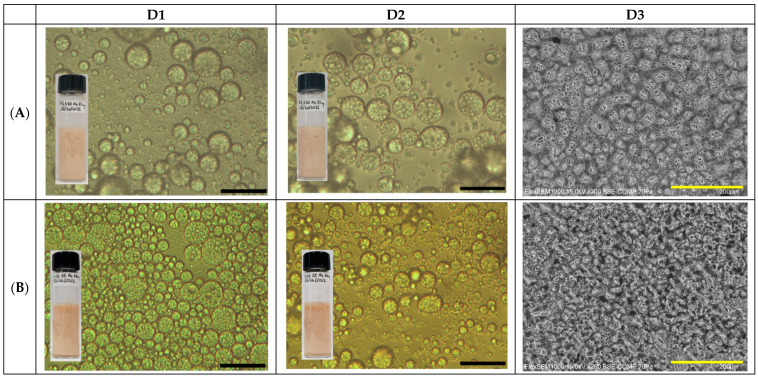

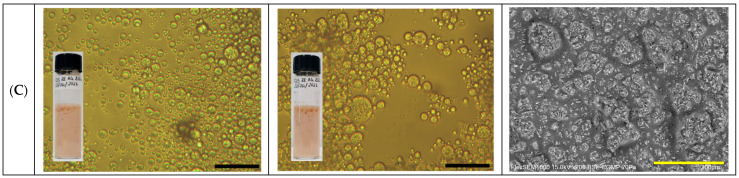
Microscopic characterization of (**A**) 05GA4_75Pe, (**B**) 05GA4_100Pe, and (**C**) 05GA4_200Pe samples. Optical microscopy images (**D1**) 1 h and (**D2**) 1 month after preparation (scale bar = 2 μm). Scanning electronic microscopy images (**D3**) 24 h after preparation (scale bar = 200 μm). Insert: visual images of different samples at corresponding times.

Added consecutively, ultrapure water, Miglyol^®^ 812N, and different amounts of GA4 powder were first gently vortexed for 60 s (1200 rpm) and successively subjected to sonication with an ultrasonic probe (400 watt) for 5 min at 25 °C under a constant N_2_ gas flow. The volume of each GA4_Pe sample was fixed at 5 mL. The effect of increasing the GA4 concentration on the stabilization of Pickering emulsions was observed at all aqueous phase/oil phase (*v*/*v*) ratios. The macroscopic aspect of the different GA4_Pes obtained from different φ_w_ values (Equation (S1)) is shown in Figure S1. From visual observation performed over time for up to 1 month of storage at 4 °C, we deduced that creaming, regardless of GA4 quantity, is always completely absent only in the samples with φ_w_ value equal to 0.5, hereafter referred to as 05GA4_XPe with X = 75,100 or 200 mg of GA4. All 05GA4_XPe samples were equally homogeneous, beige or yellow in color, and found to be oil-in-water emulsions, as confirmed by their good dispersion in water in the drop tests. Moreover, light microscopy images revealed the presence of multiple droplets (W/O/W) in all 05GA4_XPes (Figure 1), also after 1 month from preparation.

One of these multiple droplets captured by the TEM image is shown in Figure S2. The large, dark area of oil containing some clearer droplets of water and with a non-spherical contour is probably due to the drying artefacts expected as a result of the applied procedure. Drop-casting, in which one drop of an aqueous dispersion of 05GA4_XPe was placed on a TEM grid and left to air dry, may have induced changes in the morphology of the oil droplet due to the evaporation of the dispersed phase, but multiple droplets may have also burst under the vacuum of TEM.

All 05GA4_XPe samples displayed stable droplets, even in the presence of a smaller amount of GA4. This indicates that surface coating provided by 75 mg of GA4 was enough effectively to surround the oil droplets and to form a coalescence-resistant oil-water interfacial coating. Different authors have reported that some basic properties of food-grade -based Pes, such as their type (single or multiple emulsion), droplet size, physical stability, and viscosity, could be strongly affected by the stabilizer concentration [40,41]. As the concentration of GA4 increased to 40 mg/mL, the droplet interface could be more densely coated by GA4 particles.

The SEM images in Figure 1 offer insights into the overall morphology of the undiluted samples 24 h after preparation. Cryo-SEM imaging reveals that undiluted Pes are in a gelled state, exhibiting a continuous three-dimensional network in which droplets are embedded within or coated by particle-based structures, similarly to what has been found for Pickering emulsion stabilized by cellulose particles, kappa carrageenan particles or gliadin/sodium caseinate nanoparticles [42,43,44]. Looking at the images in Figure 1D3 (A–C), a qualitative inspection seems to suggest that, even if tested samples show a rather similar morphology, changes in microstructure occur at different powder contents. It is also worth noticing that a clear and systematic dependence on concentration, typical of similar systems, like gliadin/caseinate-based emulsions [44], is not appreciable in the present case. 

**Figure 2:**
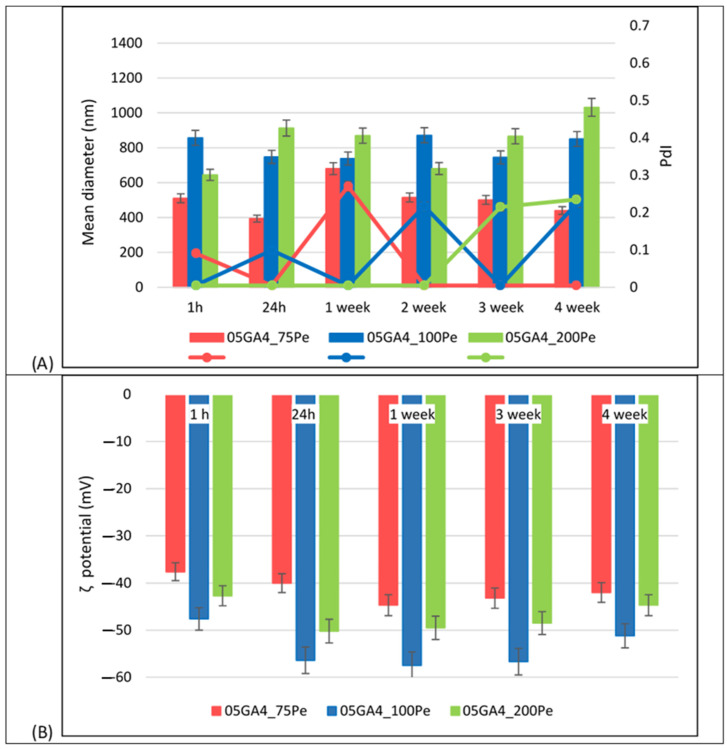
(**A**) Mean droplet diameter and PdI; (**B**) ζ potential at 25 °C of 05GA4_XPe samples acquired during storage (at 4 °C) after preparation.

In Figure 2 the mean droplet size, zeta potential, and PdI of different 05GA4_XPe samples are shown.

The droplet size distribution of 05GA4_100Pe was monomodal for up to 1 month of storage at 4 °C after preparation (Figure S3). The PDI value ranged between 0.005 and 0.225, confirming that its droplet size distribution was narrow and homogeneous throughout the storage period. Despite never exceeding a value of 0.3, a different trend of the PdI value was observed in the other two samples. In fact, the PdI of the 05GA4_200Pe sample does not show any variation for up to 2 weeks after preparation, while in the 05GA4_75Pe sample, a constant and low PdI value was measured starting from the second week. As shown in Figure 2A the mean droplet diameter of 05GA4_100Pe (850 nm) remained almost similar to that measured just 1 h after preparation, whereas some variation occurred in the other two formulations, slightly more sensitive in 05GA4_200Pe. Furthermore, an increase in droplet size was measured when the concentration of GA4 was increased beyond 20 mg/mL. This could be due to the surplus addition of stabilizer in the 05GA4_200Pe formulation, which, in turn, entails an excessive agglomeration of it around emulsion droplets, resulting in the possible formation of multilayers/aggregates at their interface for the likely excess of GA4 not adsorbed. Similar results have also been reported for Pickering emulsions stabilized by resistant starch or food-grade gelatin nanoparticles using ultrasonication [41,45].

The ζ-potential value of all 05GA4_XPe samples was higher than that of the processed GA4 particles (−13.3 ± 0.8 mV, *p* < 0.01) [11]. This significant increase in negative surface charge might be attributed to the amount of bioactive miscellaneous derived from GA4 powder and around to the oil droplets surface, compared to what was available in their simple dispersion in the aqueous phase. Usually, a high interfacial charge implies a good stability of Pickering emulsions [41], whereas coalescence, flocculation, or aggregation of oil droplets can occur with lower ζ-potential values. The measured absolute ζ-potential values higher than 30 mV are indicative of a strong electrostatic stabilization of oil droplets, as well as physical stabilization by the GA4 powder. Therefore, we believe that the good long-term stability of 05GA4_XPe samples can be attributed not only to the irreversible adsorption of GA4 particles at the oil droplet surface but also to the strong electrostatic repulsion induced by the high surface potential of dispersed oil droplets. In fact, even after 30 days of storage at 25 °C, the ζ-potential values of all 05GA4_XPe formulations were found to be negative, falling within the range of ≈−37.6 mV to ≈−57.5 mV (Figure 2B). Certainly, the behavior of GA4 powder at the oil–water interface is very important to understanding the origin of its emulsifying and stabilizing ability. Nonetheless, its physical arrangement and behavior at the interface are undoubtedly difficult to assess at the moment. In the future, a CSM (Confocal Scanning Microscopy) characterization of 05GA4_XPe systems is planned to provide further information on the role played by the different bioactive components of GA4 in structuring the aqueous phase surrounding the oil droplets.

All 05GA4_XPe samples showed a gel-like appearance, probably due to the high viscosity conferred by the relatively lofty oil content and by the structuring action of ginger particles, which make the droplets more rigid. Interestingly, no significant variation in the zeta potential value was observed over time in 05GA4_75Pe, unlike the other two samples, for which, however, increased values were observed in the presence of a higher quantity of GA4, as it should be [46]. 

**Figure 3:**
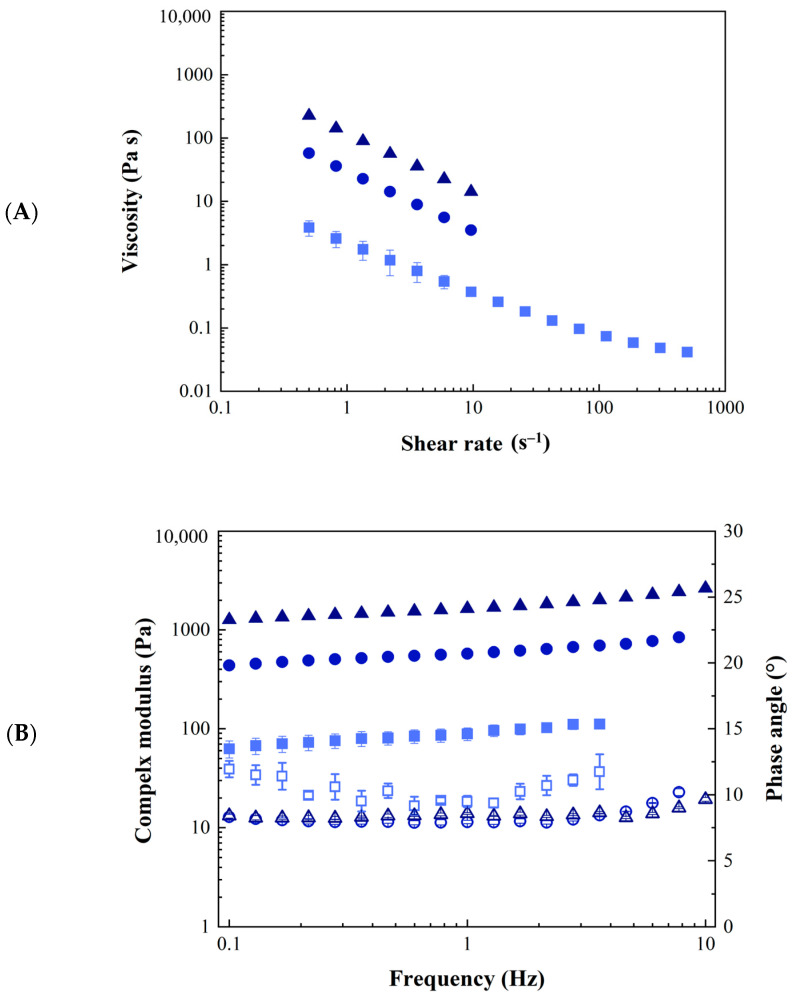
Rheological characterizations of Pickering emulsions (05GA4_75Pe, square; 05GA4_100Pe, circle; 05GA4_200Pe, triangle) at 25 °C: (**A**) viscosity; (**B**) complex modulus (closed symbols) and phase angle (open symbols).

The viscosity (*η*) of 05GA4_XPe samples as a function of shear rate is shown in Figure 3A. All samples exhibit shear thinning behavior, which is typical for such heterogeneous materials [44,47], with a linear trend in a log–log scale, which is typical of systems exhibiting a power law trend; for sample 05GA4_75Pe, a deviation from this trend is observed at high shear rates (approximately greater than 100 s^−1^), suggesting the potential onset of a Newtonian plateau. It was not possible to investigate the behavior at higher shear rates, owing to experimental issues such as slippage phenomena and edge fracture. As can be seen from the plot, an increase in the amount of ginger powder in the sample led to a significant increase in the viscosity (more than linear), in agreement with the results obtained for similar systems [44,47]. The results of frequency sweep tests carried out on the samples are reported in Figure 3B. The dynamic oscillatory behavior of samples is expressed in terms of complex modulus (G*) and phase angle (*δ*).

Similarly to the viscosity, the complex modulus (G*), that could be considered an estimate of material “consistency”, increases more than linearly with increasing powder concentration, as reported in the literature for different types of Pickering emulsions [44,47].

All samples show solid-like behavior; indeed, independently from powder concentration, phase angle (*δ*) is close to 10° indicating a weak gel behavior, in agreement with the results on similar systems [44]. Sample 05GA4_75Pe shows phase angle values slightly higher than those of 05GA4_100Pe and 05GA4_200Pe, suggesting the presence of a less structured system, probably owing to the lower powder content. The flow data of sample 05GA4_75Pe were fitted using the Sisko model, whereas for the other two samples, the power law model was used. All the results of the dynamic tests were fit with a weak gel model. The fitting parameters are reported in Table 1. Both the consistency coefficient (*K*) and the gel strength (*A*) notably increase with increasing ginger powder concentration. The flow index (*n*) decreases from approximately 0.19 to 0.05 with an increase in the powder concentration from 15 mg/mL to 20 mg/mL, indicating more non-Newtonian behavior and a stronger dependence on the shear rate, which suggests a more relevant breakage of the structure as a consequence of the increase in the shear rate. A further increase in the powder concentration does not involve changes of the flow index, as confirmed by ANOVA analysis. As far as the network extension (*z*), no significant dependence on powder concentration was observed.

### 2.3. Preparation and Characterization of DOX-Loaded GA4_Pes

**Figure 4:**
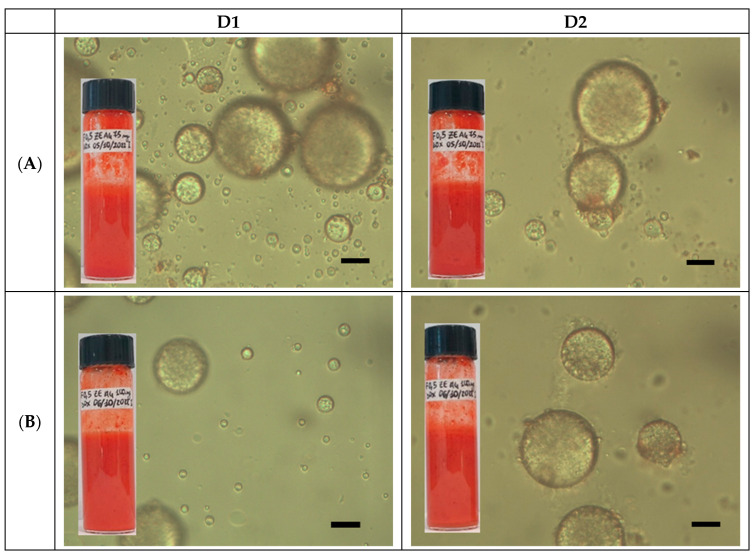
Optical microscopy images of (**A**) 05GA4_75Pe@DOX and (**B**) 05GA4_100Pe@DOX samples (**D1**) 1 h and (**D2**) 1 month after preparation (scale bar = 0.5 μm). Insert: visual images of different samples at corresponding times.

For doxorubicin-loaded GA4-based Pickering emulsions, only the 1:1 oil/water ratio was selected, and only 05GA4_XPe@DOX samples prepared at GA4 concentrations of 15 mg/mL and 20 mg/mL and containing a DOX concentration of 2.5 mg/mL were initially studied. Although a DOX dissolution step in water at 40 °C was first required, 05GA4_XPe@DOX samples were prepared in the same way as GA4_Pes. Characterization studies on 05GA4_XPe@DOX were undertaken as described above. For both samples, a dense emulsion layer was rapidly distinguished after preparation. The emulsion phase filled the entire vials, at least during the first month of storage at 4 °C (Figure 4), and no phase separation was discerned. A dark-red O/W emulsion was obtained for both 05GA4_XPe@DOX samples, as confirmed by drop tests.

**Figure 5:**
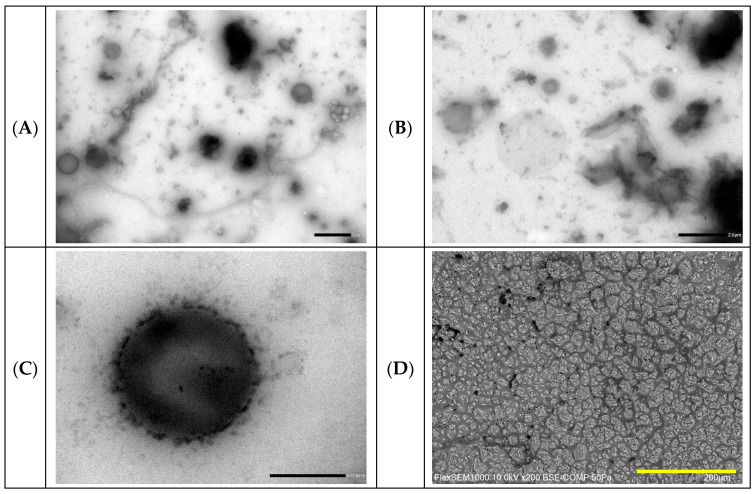
(**A**–**C**) TEM images (scale bar: (**A**,**B**) = 2 μm; (**C**) = 500 nm) and (**D**) SEM image of 05GA4_75Pe@DOX (scale bar = 200 μm).

The TEM images of 05GA4_75Pe@DOX acquired 24 h after preparation (Figure 5) clearly show that the GA4 particles were sometimes adsorbed onto the surface of the emulsion droplet differently. Furthermore, GA4 appeared to be swollen or aggregated onto some droplets, with most of these clusters closely associated with the surface. The droplet interface appeared to be covered mainly by a continuous network of fused or aggregated particles rather than by individual and distinguishable GA4 particles, as observed in a single droplet captured in the unloaded 05GA4_75Pe sample (Figure S4). It should be emphasized, however, that coverage by Pickering particles may not be complete, despite producing stable emulsions, as long as the layer of adsorbed particles forms a rigid network. In TEM images (Figure 5), multiple and double droplets appear, with evident contrast differences between the dark and light areas, probably as a function of the differentiation in the molecular masses between the droplet surface area compared to the internal one. The latter, in turn, may be the result of the entrapment of a single droplet or large number of smaller droplets, either with or without DOX [48,49].

Undoubtedly, DOX loading does not modify the stability of the unloaded 05GA4_XPe analogs, although it leads to slight change in the morphology and droplet average size. Indeed, even in doxorubicin-loaded 05GA4_XPes, the presence of numerous very small droplets is clearly distinct within some larger droplets, as shown in optical images (Figure 4). Some small droplet agglomerates with red cores are also observed to be adsorbed on multiple larger droplets. The red color is due to the doxorubicin inside the dispersed aqueous phase entrapped in the smaller droplets. The similar morphology of DOX-loaded and unloaded 05GA4_75Pe samples was confirmed by SEM (Figure 5D).

**Figure 6:**
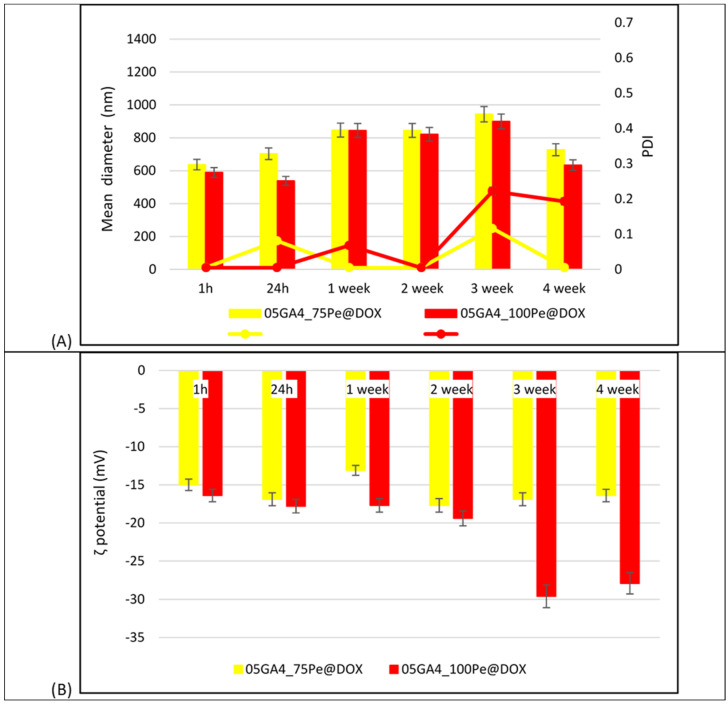
(**A**) Mean droplet diameter and PdI; (**B**) ζ potential at 25 °C of 05GA4_XPe@DOX samples acquired during storage time (at 4 °C) after preparation.

In Figure 6A the average droplet sizes, PdI, and ζ-potential values determined at 25 °C during the entire storage period (4 °C, 4 weeks) are shown. These measures were acquired on 05GA4_XPe@DOX aqueous dispersions diluted and vortexed for 5 s to ensure that the light scattering intensity was within the range of instrument sensitivity. Although moderately larger for 05GA4_75Pe@DOX, the average droplet sizes were less than 750 nm for both samples 1 h post preparation, as well after 24 h. Unlike the unloaded sample, the observed decrease in the droplet average diameter of the 05GA4_100Pe@DOX sample was probably due to the greater number of negative charges, owing to the greater amount of stabilizer, which could create more ionic interactions by attracting and neutralizing the positive charges in DOX [50]. A stronger electrostatic attraction with DOX can influence the hydrophilic nature of GA4, thereby inducing variation in GA4 particles adsorption, in addition to reducing the possible formation of non-adsorbed multilayers/aggregates on the surface of the oil droplets, as previously hypothesized for unloaded 0.5GA4_XPe. After 28 days, no significant change in average hydrodynamic diameters was individuated in either 05GA4_XPe@DOX samples, whereas the value of PdI for only 05GA4_100Pe@DOX was increased to 0.2. The measured ζ-potential values for 05GA4_100Pe@DOX confirm the hypothesis of specific electrostatic interactions between GA4 and the drug, which appeared to change over time. Instead, even though they remained constant, on average, over time (Figure 6B), 05GA4_75Pe@DOX ζ–potential values were lower than those of similar unloaded samples (Figure 2B), although without affecting stability deeply. Furthermore, 0.005 ≤ PdI ≤ 0.100 indicated a homogeneous and monomodal droplet size distribution that was steady throughout the storage period. Considering that the stability of the dimensional values, i.e., PdI and ζ-potential, are fundamental in a therapeutic formulation of DOX, here, we preferred to present the rheological characterization of only 05GA4_75Pe@DOX, postponing the presentation of that of the other 05GA4_XPe@DOX samples to a subsequent work that will report on the effects of different contents of GA4 stabilizers over time and at different temperatures on their rheology. This study is ongoing.

**Figure 7:**
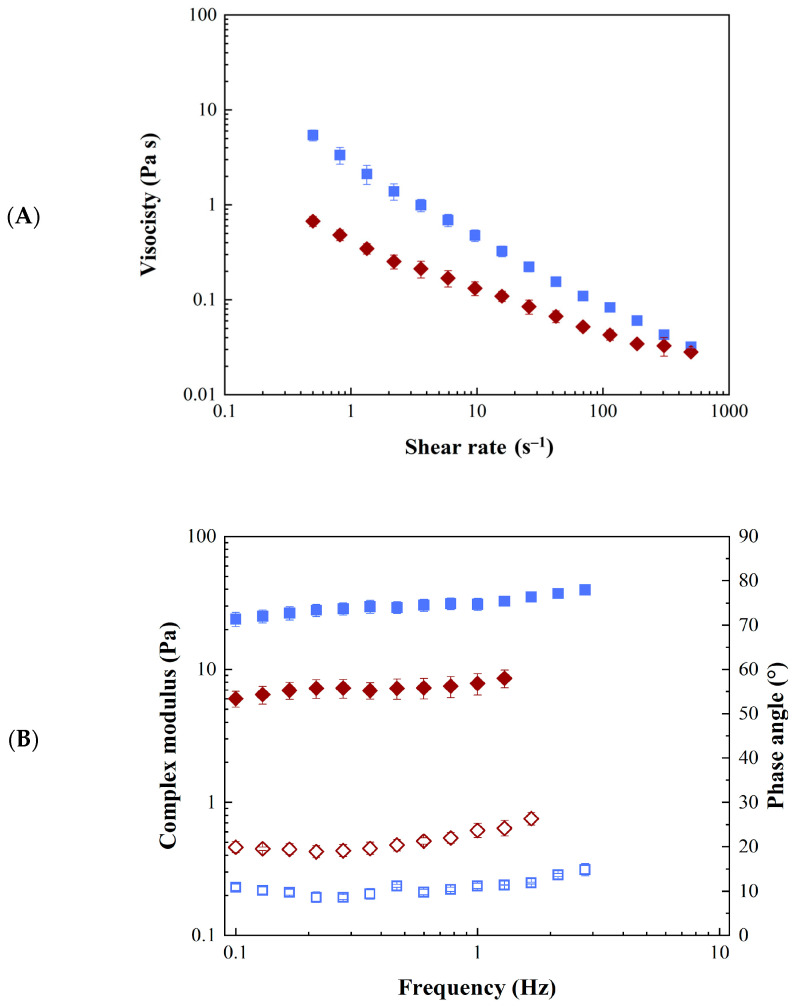
Rheological characterizations of unloaded and loaded Pickering emulsions (05GA4_75Pe, square; 05GA4_75Pe@DOX, diamonds) at 37 °C: (**A**) viscosity; (**B**) complex modulus (closed symbols) and phase angle (open symbols).

The comparison of rheological properties of sample 05GA4_75Pe and sample 05GA4_75Pe@DOX at 37 °C is reported in Figure 7. Both samples exhibited shear thinning behavior (Figure 2A), in agreement with literature results [44,47], and the onset of a Newtonian plateau at a high shear rate; the addition of doxorubicin yielded a reduction in viscosity more evident at low shear rates. This finding is in contrast with the results reported by other authors for non-structured emulsions (O/W microemulsion) loaded with doxorubicin, for which increases in viscosity and non-Newtonian behavior were found to be a consequence of doxorubicin addition [51,52]. Greater differences in the rheological behavior of samples 05GA4_75Pe and 05GA4_75Pe@DOX can be observed in Figure 7B.

Specifically, from dynamic oscillatory tests, lower values of G* and higher values of *δ* were found in the whole frequency window for sample containing doxorubicin. Despite both samples still behaving as weak gel material [44], the addition of doxorubicin led to a doubling of the phase angle, suggesting a relevant decrease of the structuring degree. This result is also in contrast with the trend found by other authors for a doxorubicin-loaded microemulsion, for which increases in *G*′ and *G*″, and, therefore, in G*, were found [51]. It is worth noticing that the data available in the literature concerning the effect of doxorubicin on rheological properties of emulsion-based systems [51,52] refer to non-structured emulsions (microemulsions) with liquid-like behavior (i.e., *G*″ > *G*′, *δ* > 45°), whereas, to the best of our knowledge, no information is available on the effect of doxorubicin on the rheology of structured systems (*δ* < 45°) like Pickering emulsions and/or emulgels. Specifically, the change of rheological properties of such a type of systems is also connected, in addition to O/W interfacial properties, to the interaction between doxorubicin and the powder in the emulsion, i.e., the ginger powder for the Pickering emulsion in the present study. The changes in the rheological properties of sample due to DOX addition found in the present work were quantified by fitting experimental data with the Sisko model (flow curve test) and weak gel model (dynamic oscillatory tests) at both 25 °C and 37 °C. Fitting parameters are reported in Table 2 with the data of sample 05GA4_75Pe at 25 °C, as reported in Table 1, providing a complete overview of the combined effect of DOX addition and temperature on the rheological properties of the system.

Specifically, concerning the flow properties, at 37 °C, the infinite shear viscosity (*η_∞_*) decreases with the addition of DOX, whereas for both 05GA4_75Pe and 05GA4_75Pe@DOX, it does not change significantly by changing temperature from 25 to 37 °C. The consistency coefficient (*K*) of 05GA4_75Pe and that of sample 05GA4_75Pe@DOX are similar at 25 °C, whereas, following the reduction due to temperature, at 37 °C, the DOX-loaded sample exhibited a lower value compared to that of the unloaded one. For 05GA4_75Pe, the flow index (*n*) was not significantly affected by temperature (approximately 0.19 at 25 °C and 0.24 at 37 °C) and, at 25 °C neither by DOX addition (approximately 0.17); however, at 37 °C, the value of n of sample 05GA4_75Pe@DOX was resulted notably higher (approximately 0.43). Considering the microstructural properties, the addition of DOX notably decreases the gel strength (*A*), which was found to decrease with temperature for sample 05GA4_75Pe, whereas for sample 05GA4_75Pe@DOX, the decrease of A due to temperature was not significant. The coordination number (*z*) was not significantly affected by DOX addition, and for sample 05GA4_75Pe, it was not affected by temperature either; however, for sample 05GA4_75Pe@DOX, an increase in *z* was found to coincide with an increase in temperature from 25 °C to 37 °C.

Overall, in light of these outcomes, it is possible to conclude that the effects of DOX addition on the rheological properties of sample 05GA4_75Pe are more relevant at 37 °C.

### 2.4. In Vitro Drug Release Analysis

There may be various causes for the failure of conventional chemotherapeutics in the treatment of skin cancer. Among these, we recall a lack of drug metabolic activation, possible apoptosis pathway alteration, and an excess drug flux to the solid tumor, all of which may induce drug resistance, sometimes associated with increases in the levels of proteins such as P-glycoprotein [53]. Hence, controlled delivery systems for targeted therapies to control drug resistance are highly desirable. However, an important goal in innovative cancer therapies is also to try to reduce adverse effects resulting from both non-targeting and drug overdose [54]. Generally, controlled drug retention and sustained release are principal items (or goals) for the optimal performance of a smart antineoplastic drug delivery system (ADDS) [55]. In this study, controlled delivery of chemotherapeutics via ginger powder-based Pickering emulsions was investigated as a new approach for potential local skin applications. Herein, the main aim was to propose novel DOX-loaded Pickering emulsions capable of uptake in cancer cells, avoiding the healthy skin cells. The DOX entrapment efficiency was 98.5 ± 3.2%, probably due to the presence of many double droplets, which favored encapsulation in the internal aqueous phase [56].

**Figure 8:**
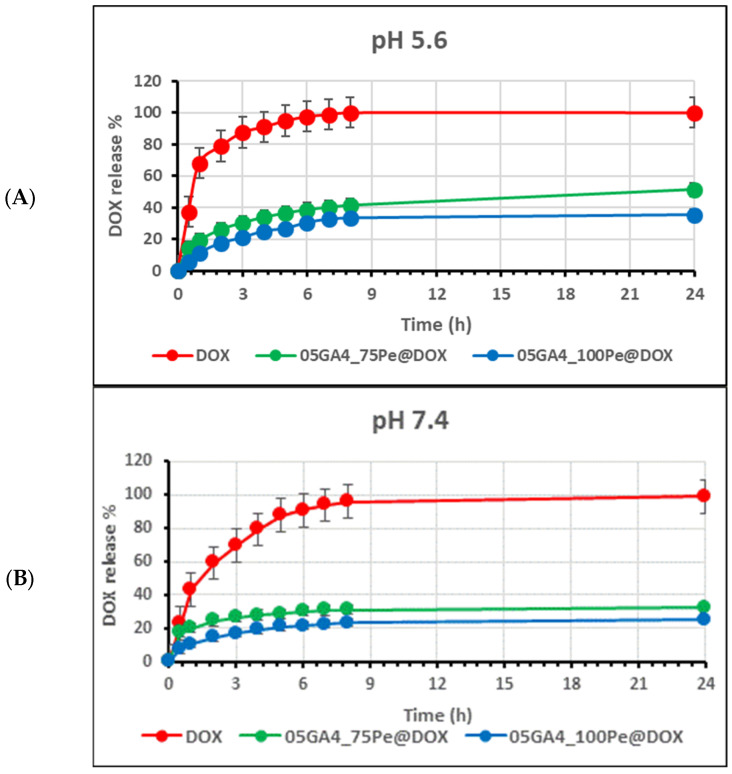
DOX release profiles from 05GA4_XPe@DOX and DOX solutions under different pH conditions (mean ± standard deviation, *n* = 3): (**A**) pH 5.6 and (**B**) pH 7.4.

In vitro release was studied using the dialysis method to quantify the cumulative % released DOX. The doxorubicin release profiles of 05GA4_XPes@DOX and control solutions were recorded over a time period of 24 h in PBS at 37 °C and under different pH conditions simulating the normal physiological pH (7.4) and the endosomal one of cancer cells (5.6) (Figure 8A,B).

The single 05GA4_XPe@DOX formulations exhibited slightly different release patterns, with no statistically significant differences evident, while they were significantly delayed and slower than those of the free DOX solutions, as found in similar W/O/W microemulsions [56]. After 4 h of incubation in PBS (pH 7.4), about 80% of DOX was released from DOX solution, while the cumulated release ratio for 05GA4_100Pe@DOX and 05A4_75Pe@DOX was up to only about 20% and 30%, respectively. After 24 h, DOX release was nearly complete from the free solution, while it remained almost unchanged from both 05GA4_XPe@DOX formulations. The release profiles of DOX from ginger powder-based Pickering emulsions were effectively delayed compared to the free DOX solution. The slow and sustained release process without any premature burst effect could be mostly driven by drug diffusion across the oil phase before reaching the O/W interfaces [37,57,58]. By adopting acidic conditions during the release test, different temporal patterns were observed, slightly more uncontrolled for the free DOX solution and less sustained for both 05GA4_XPe@DOX emulsions. In PBS 5.6, after only 6 h, 100% drug release from the free solution had already occurred, compared to only 40% from 05GA4_75Pe@DOX. This can be attributed to the easier protonation of functional groups in both DOX and GA4 at lower pH values and the consequent reduction in hydrophobic interactions between the drug and GA4. It is possible that the pH 5.6 condition favored the release of DOX entrapped in the droplet O/W interface, as also already reported in the literature [54]. We believe that, although certainly promising, the limited increase in DOX release after 24 h may be influenced by the various bioactive components present in ginger, which are probably also sensitive to pH variations like DOX. In any case, the reported values, although far from desirable, are completely in line with those that other similar drug delivery systems present after 24 h [35]. It should be noted that no destabilization phenomena, such as coalescence, were observed in the dialysis bags of either samples at the end of the release experiments under different pH conditions. No significant shifts were observed in the DOX UV-Vis spectrum in either the UV (252 and 233 nm) or visible (480 nm) region. This leads us to deduce that DOX does not show signs of instability, at least up to 24 h. The slower DOX release with increasing GA4 concentration could be indicative of an increase in viscosity in the oil phase, as well as, perhaps, the presence of internal droplets more resistant to breakage or coalescence due to a stronger O/W interface. These release experiments clearly indicate that DOX release from 05GA4_XPe@DOX systems was time-dependent, as well as pH-specific. Although further investigations should be performed to elucidate the release mechanism, the 05GA4_XPe@DOX pH-sensitive behavior confirmed their potential as DDSs, which could minimize the exposure of healthy cells to DOX while allowing for the accumulation of therapeutic drug into cancer tissues. Moreover, considering that under our experimental conditions, we used a somewhat higher concentration than the dose required for a therapeutic effect, we can say that 05GA4_75Pe@DOX shows great potential as an alternative smart DDS for in vivo application of DOX. Stability and photodegradation studies of DOX trapped in 05GA4_XPe@DOX are already underway in order to better understand the role of the bioactive substances of ginger in the release mechanism of DOX.

### 2.5. In Vitro Cytotoxicity Studies

**Figure 9:**
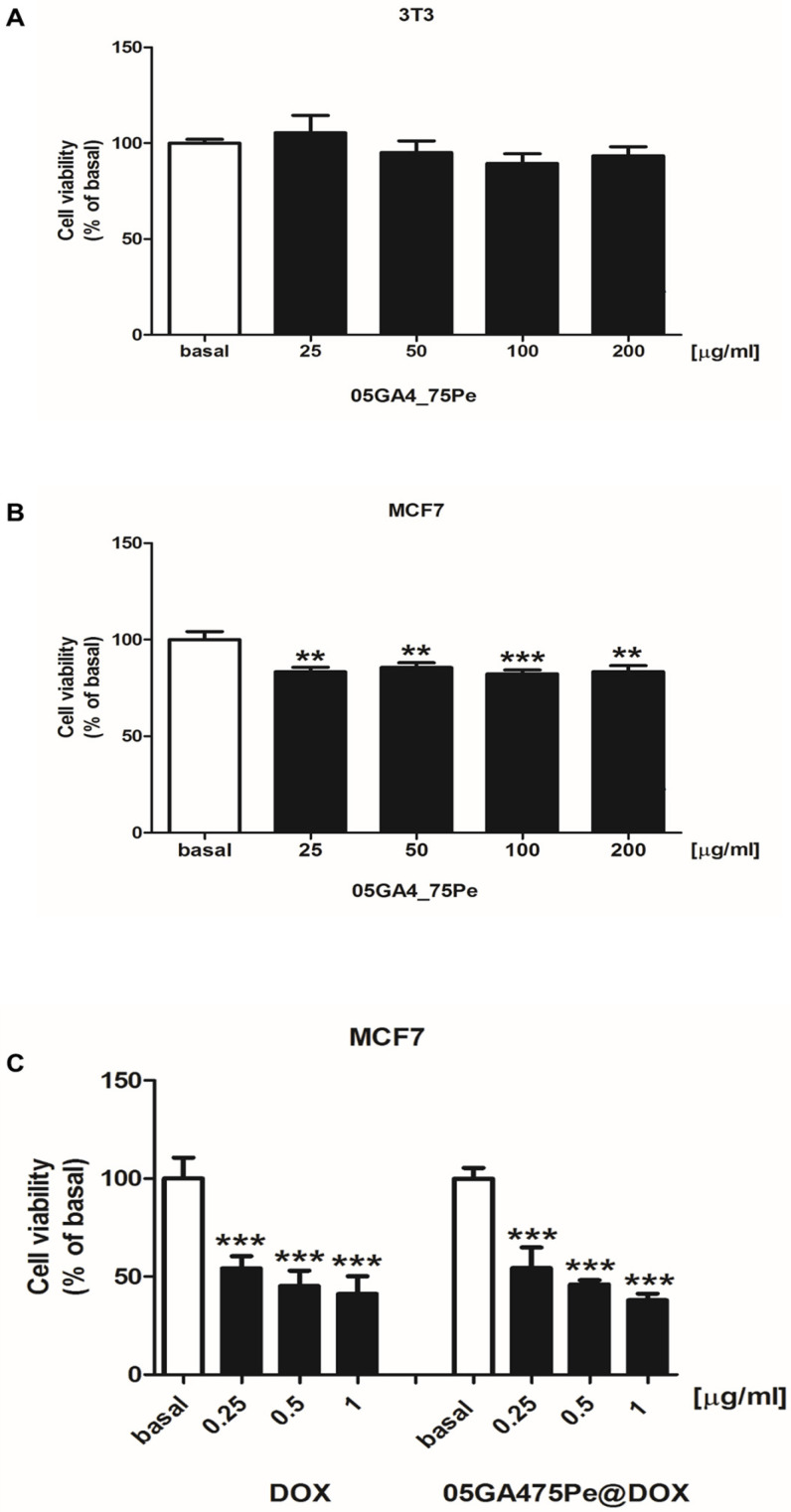
Effects of 05GA4_Pe75 on 3T3 and MCF7 (**A**,**B**) and of DOX and 05GA4_Pe75@DOX MCF7 cell viability (**C**). (**A**,**B**) 3T3 and MCF7 cells were untreated (basal) or treated for 72 h with increasing doses of 05GA4_Pe75 (25, 50, 100, and 200 μg/mL). (**C**) MCF7 cells were untreated (basal) or treated for 72 h with increasing doses of DOX (0.25, 0.5, and 1 μg/mL) or 05GA4_Pe75@DOX (0.25, 0.5, and 1 μg/mL). Cell viability was assessed by MTT assay. Results are expressed as mean ±SD of three independent experiments, each performed in quadruplicate (**, *p* < 0.001; ***, *p* < 0.0001; compared to basal).

The effects of the unloaded DOX 05GA4_Pe75 sample on immortalized 3T3 murine fibroblasts and MCF7 human breast cancer cell viability were evaluated using different dilutions corresponding to GA4 concentrations ranging from 50 to 200 µg/mL. Results showed that this sample had no toxic effect on 3T3 cells (Figure 9A), with a slight reduction of about 20% of MCF-7 cell viability (Figure 9B). Interestingly, for the doxorubicin-loaded emulsion, 05GA4_Pe75@DOX, a cytotoxic effect similar to that of the same doses of free DOX was observed in the MCF7 cell line (Figure 9C) after 72 h of treatment. By comparing the IC_50_ values for the MCF7 cell line between 05GA4_Pe75@DOX and free DOX, we obtained similar results, making the efficacy of 05GA4_Pe75@DOX clinically relevant (Table S2). Exposure to 05GA4_Pe75@DOX for 72 h should be more than sufficient to allow for nearly complete release of the internalized doxorubicin, considering that our results showed 50% drug release at pH 5.6 after only 24 h. 

## 3. Materials and Methods

### 3.1. Materials

Dried white ginger powder (GA4) obtained by conventional agricultural practices was purchased from a local market and used as received, without any pre-processing of delipidation, sieving, grinding, or milling. Miglyol^®^ 812N derived from vegetable-based raw materials (coconut/palm kernel) was kindly provided by Eigenmann & Veronelli S.p.A., Rho (Milano), Italy. Miglyol^®^ 812N—essentially, a mixture of medium chain triglycerides (30–45% capric acid and 50–65% caprylic acid) with water solubility lower than 0.01 g/L at 20 °C—is fully compliant with the requirements of the current Ph. Eur. Distilled water was obtained by purification using an ultrapure MilliQ Direct Type 1 water system (Millipore, Singapore, 18.2 MΩcm). Doxorubicin (United States Pharmacopeia (USP) Reference Standard) was supplied by Sigma Chemical Co., Milan, Italy.

### 3.2. Preparation of Ginger Powder-Based Pickering Emulsions (GA4_Pes)

Ginger powder-stabilized Pickering emulsions (**GA4_Pes**) with various **GA4** concentrations (15, 20, and 40 mg/mL) were prepared as previously described [11]. Briefly, all formulations were prepared using a 3 mm ultrasonic probe (Ultrasonic UP400S, Hielscher, Teltow, Germany) for 5 min at an amplitude of 100% and a duty cycle of 1 s. The frequency of the instrument was 24 KHz. The ultrasonic treatment was carried out at a controlled temperature of 25 °C in an ice bath after gentle agitation of the sample by vortex (1 min. 1200 rpm). The volume of each **GA4_Pe** sample was fixed at 5 mL. Once prepared, the GA4_Pe samples with all oil–water ratios (*v*/*v*) were subsequently stored at 4 °C until further use.

### 3.3. Preparation of Ginger Powder-Based Pickering Emulsions Loading Doxorubicin GA4_Pe@DOX

For doxorubicin-loaded **GA4_Pes**, only the 1:1 oil/water ratio was studied. Doxorubicin (**DOX**) was dissolved in ultrapure water (at pH 6.8) at a concentration of 5 mg/mL by magnetic stirring for 20 min in the dark to form a red transparent solution before preparing the **GA4_Pes@DOX** at different concentrations of **GA4** (15 mg/mL and 20 mg/mL). Then, doxorubicin-loaded GA4-based Pickering emulsions (**GA4_Pe@DOX**) were prepared according to the method described in Section 3.2. All emulsions were stored at 4 °C until further use.

### 3.4. Physicochemical Characterization

#### 3.4.1. Stability and Type

The physical stability of the ginger powder-stabilized Pickering emulsions was monitored as a function of the storage time (from 1 h after preparation up to 1 month) in order to determine whether any phenomena of sedimentation, coalescence, flocculation, and/or cream formation remained stable. Samples were stored at 4 °C in a glass vial sealed with a cap to prevent them from drying, and their macroscopic appearance was periodically analyzed by visual inspection (bottle test) of the volume fraction of water and/or oil resolved from the emulsion layer.

As a key Pickering emulsion indicator, the emulsification index (EI) is usually evaluated to assess the emulsification capability [59]. Fresh prepared GA4_Pes were placed at 4 °C for 24 h and successively at room temperature (RT) for 24 h to reach a stable state. Then, the EI of the **GA4_Pes** after storage was calculated by Equation (1):

EI (%) = H_e_/H_t_ × 100 (1)

where H_e_ is the total height of the emulsion layer and H_t_ is the total height of the liquid phase (corresponding to 5 mL of sample). The same stability tests and EI measurements were applied for doxorubicin-loaded **GA4_Pes**.

The type of Pickering emulsions obtained from ginger powders was checked by a drop test immediately after emulsification. Drops of DOX-loaded or unloaded **GA4_Pes** were dripped into pure Miglyol^®^ 812 N and pure water: if the drops readily dispersed in Miglyol^®^ 812 N (or in water) and not in water (or in Miglyol^®^ 812 N), the Pickering emulsion was of w/o (or o/w) type. In addition, if a drop of oil, mixed within the DOX-loaded or unloaded **GA4_Pe**, moved towards the top (or bottom) of the emulsion, water (or oil) was the continuous phase.

#### 3.4.2. Droplet Size Analysis and ζ-Potential Measurements

The droplet size distribution and polydispersity index (PdI) of **GA4_Pes** were assessed 1 h after preparation and periodically during the storage time by dynamic light scattering (DLS) using a 90Plus Particle Size Analyzer (Brookhaven Instruments Corporation, Holtsville, NY, USA) at different temperatures. The polydispersity index (PdI) discloses the emulsion quality, and values lower than 0.3 are indicative of homogenous and monodisperse size populations and high-quality samples [60]. All of the measurements were carried out in triplicate using 1.45, 1.59, and 1.33 as the refractive index for ginger particles, Miglyol^®^ 812 N, and water, respectively. The ζ potential of **GA4_Pes** was measured at 25 °C 1 h after preparation and periodically during the storage time using a NanoZS Zetasizer instrument (Malvern Instruments Ltd., Worcestershire, UK). The mean value and standard deviation of the ζ potential of each **GA4_Pe** were calculated from at least three measurements. All GA4_Pe samples were suitably diluted with ultrapure water (1:100), avoiding multiple scattering effects during DLS measurements of droplet size and ζ potential.

The same methodology used to analyze the particle size distribution profile, hydrodynamic diameter, PdI, and ζ potential was applied for doxorubicin-loaded **GA4_Pes**.

#### 3.4.3. Optical Microscopy

A Motic 300POL optical microscope equipped with a Moticam 2.0 digital camera was used to assess the morphology of DOX-loaded and unloaded **GA4_Pes**. For better microstructure imaging, it was always necessary to dilute every sample with ultrapure water (1:5) before optical observation. To avoid any damage of **GA4_Pes** morphology from droplet coalescence for shear effects, a hand-made glass sample holder realized with spacers (0.1 mm) laterally fixed between the specimen slide and the cover slip was set up. A drop of diluted **GA4_Pe** sample was carefully deposited on a specimen slide and safely covered by a cover slip. Microscopic analysis at room temperature was conducted periodically during storage at 4 °C, from 1 h to 1 month after the emulsification process.

#### 3.4.4. Cryo-Scanning Electron Microscopy (Cryo-SEM)

With the aim of visualization, the direct morphology of DOX-loaded and unloaded **GA4_Pe** samples was also investigated using scanning electron microscopy investigations of undiluted samples under cryoscopic conditions with a FlexSem 1000 II (Hitachi, Tokyo, Japan). Each sample was frozen at −35 °C using a Peltier cooling stage (Coolstage, Deben UK, Suffolk, UK). Micrographs were acquired at 200× using an accelerating voltage of 10–15 kV and a pressure of 50–70 Pa (low vacuum). Micrographs were obtained from the processing of the backscattered electron (BSE) signal, and no metallization of the sample surface was used for the observations.

#### 3.4.5. Transmission Electron Microscopy (TEM)

The morphology of DOX-loaded and unloaded **GA4_Pe** samples was also evaluated by TEM micrographs using a JEOL 1400 TEM electron microscope (Jeol 1400 Plus electron microscope, JEOL Ltd., Milan, Italy) operating at an accelerating voltage of 80 kV. For TEM imaging, all samples were diluted in ultrapure water (1:5), then dried at room temperature before microscopic observation. All observations were also performed at room temperature.

#### 3.4.6. Rheological Characterization and Analysis

Rheological characterization was carried out using a HAAKE MARS III rotational rheometer (Thermo Fisher Scientific, Waltham, MA, USA) equipped with a parallel plate geometry (diameter of 20 mm and gap of 1.2–1.5 mm). Flow curves were determined in the shear-rate range of 0.5–500 s^−1^. For samples **05GA4_100Pe** and **05GA4_200Pe**, given the high consistency, sandblasted plates (diameter of 20 mm and gap of 1.5) were used to limit slippage phenomena. Nevertheless, experimental issues were observed at high shear rates, measurements unreliable at values greater than 10 s^−1^.

Frequency sweep tests were carried out between 0.1 and 10 Hz under linear viscoelastic conditions, as determined with preliminary stress sweep tests.

The viscosities of samples 05GA4_75Pe and 05GA4_75Pe@DOX were fit using the Sisko model [61] (Equation (2)).

(2) $$\eta ={\eta_{\infty }+K\;\dot{\gamma }}^{n-1}\;$$

where η is the viscosity (in Pa s), $\eta_{\infty }$ is the infinite shear viscosity (in Pa s), K is the consistency coefficient (in Pa s^n^), and n is the flow index (dimensionless).

The viscosity data of samples 05GA4_100Pe and 05GA4_200Pe were correlated with the shear rate though the power law model [48] (Equation (3)).

(3) $$\eta ={K\;\dot{\gamma }}^{n-1}\;$$

where η is the viscosity (in Pa s), K is the consistency coefficient (in Pa s^n^), and n is the flow index (dimensionless). The results of the frequency sweep tests were fit using the weak gel model [62] (Equation (4)).

(4) $$G^{*}=\sqrt{{\left(G^{\prime }\right)}^{2}+{\left(G^{\prime \prime }\right)}^{2}}=A\omega^{\frac{1}{z}}\;$$

where $G^{*}$ is the complex modulus (In Pa), ω is the angular frequency (In Hz), A is a measure of the gel strength (in Pa s^1/z^), and z is the coordination number or network extension (dimensionless). All rheological tests were carried out at 25 °C. Further frequency sweep tests and flow curve tests were carried out at 37 °C for the doxorubicin-loaded sample and reference sample, i.e., the Pickering emulsion with the same ginger powder concentration of the Pickering emulsion containing doxorubicin. All measurements were performed in triplicate, and the results were averaged.

#### 3.4.7. Statistical Analysis

Data fitting and statistical analysis were performed with Origin Pro software (Version 2023b; OriginLab Corporation, Northampton, MA, USA). A one-way ANOVA test was used to compare the mean values of rheological parameters (consistency coefficient, flow index, gel strength, and coordination number) of Pes under different GA4 contents; differences were considered significant for *p* < 0.05, and Fisher’s test was used. A two-way ANOVA test was used to compare the mean values of rheological parameters (infinite shear viscosity, consistency coefficient, flow index, gel strength, and coordination number) of Pes with and without DOX at different temperatures.

### 3.5. DOX Entrapment Efficiency in GA4_Pes

To obtain the DOX entrapment efficiency in the DOX-loaded **GA4_Pes**, 1 mL of each sample was dialyzed (12–14 kDa cutoff, Spectrum Laboratories, Inc., Rancho Dominguez, CA, USA) against 50 mL of PBS (pH 7.4) for 1 h under constant stirring [63]. The amount of free DOX in PBS was spectrophotometrically quantified by reading the absorbance at 481 nm with a JASCO V-530 UV-vis spectrometer (JASCO Corporation, Tokyo, Japan) and by using a calibration standard curve of DOX in PBS (pH 7.4) calculated in the range of 0.6–40 μg/mL [y = 16.787x + 0.0066 with coefficient regression R^2^ = 0.9998]. All free DOX determinations were performed in triplicate, and the results were expressed as mean ± SD. The following formula was employed to calculate the *EE*% (Entrapment Efficiency) (Equation (5)).

(5) $$EE\;\%=\frac{Total\;drug\;loaded-untrapped\;drug}{Total\;drug\;loaded}\times 100\;$$

### 3.6. In Vitro Drug Release Studies

The drug release profiles of DOX-loaded **GA4_Pes** were assessed by the dialysis method. We analyzed DOX release from **05GA4_XPE@DOX** and plain solutions of DOX (2.5 mg/mL) in PBS 0.01 M (pH 5.6 and pH 7.4). An aliquot of the DOX-loaded GA4_Pe sample (0.8 mL) or of solutions (1 mL) equivalent in terms of DOX amounts was transferred to a dialysis bag. The sealed minibags were immersed, floating, in a 50 mL release medium (PBS a pH 5.6 and 7.4) contained in orbital shakers under constant stirring at 200 rpm at 37 °C in the dark. Sampling (2 mL) was performed at predetermined time intervals up to 24 h. To maintain sink conditions, the dialyzed volume was supplemented with 2 mL fresh release medium after each withdrawal. The amount of released DOX was directly analyzed by UV–Vis spectrophotometry at 481 nm. The concentration was determined with reference to the different calibration curves of DOX in PBS at pH 7.4 and pH 5.6, (the standard curve of DOX at pH 5.6 is y = 19.215 x + 0.0093 with a regression coefficient of R^2^ = 0.9995). Release profiles were calculated in terms of cumulative release (%) over incubation time from individual experiments on triplicate samples. The in vitro drug release profile of DOX solutions was used as a control.

### 3.7. Cell Cultures

The human breast cancer MCF-7 cell line and immortalized murine 3T3 fibroblasts were purchased from the American Type Culture Collection (ATCC) (Manassas, VA, USA). MCF-7 cells were maintained in Dulbecco’s Modified Eagle Medium/F12 (DMEM/F12) supplemented with 5% fetal bovine serum (FBS) medium, 1% L-glutamine (L-Glu), and 1% penicillin/streptomycin (P/S) (Sigma-Aldrich, Milano, Italy). 3T3 cells were maintained in DMEM with phenol red supplemented with 10% bovine calf serum (BCS), 1% L-Glu, and 1% P/S. All cells were maintained at 37 °C in a humidified atmosphere of 95% air and 5% CO_2_ and were screened periodically for mycoplasma contamination.

### 3.8. MTT Cell Viability Assay

The in vitro cell viability of 3T3 or MCF7 exposed to increasing doses of 05GA4_Pe75 (25, 50, 100, and 200 μg/mL) (3T3 and MCF7 cells), DOX, or 05GA4_Pe75@DOX (0.25, 0.5, and 1 μg/mL) (MCF7 cells) was measured using an MTT assay as previously described [64]. Briefly, cells were seeded in a 48-well plate, allowed to grow for 48 h, then treated for 72 h, as indicated. After treatment, fresh MTT (Sigma), resuspended in PBS, was then added to each well (final concentration of 0.5 mg/mL). After 2 h of incubation, cells were lysed with 200 μL of DMSO, and optical density was measured at 570 nm using a Synergy H1 Hybrid Reader (Bioteck S.p.A, Milan, Italy).

## 4. Conclusions

Key findings gathered throughout this study support the efficacy of engineering (designing) a biocompatible ginger powder-based Pickering emulsion to be used as a therapeutic platform for topical doxorubicin administration. Ginger nanoparticles are sustainable, biocompatible, and biodegradable and display an excellent emulsifying capability, also at low concentrations. GA4_Pe answers the main prerogatives, allowing for a controlled topical chemotherapy: (1) stable over weeks, (2) multiple-droplet W/O/W morphology for good target-site selectivity, (3) uniform droplet size distributions, (4) loadable with doxorubicin dose as recommended by the medical scientific community, and (5) pH-sensitive and sustained release of the antineoplastic drug to extend exposure to cancer cells. Efficient entrapment of anticancer drug doxorubicin into GA4_Pe was simply performed by mixing the doxorubicin aqueous phase with Miglyol^®^ 812 N and ginger powder by one-step emulsification procedure. The morphological, dimensional, and rheological properties, as well as the stability of GA4_Pe@DOX emulsions were not deeply changed by entrapped DOX. Ginger powder-based Pickering emulsions had no cytotoxic effect in normal 3T3 fibroblasts, while a small inhibitory effect was observed in MCF-7 breast cancer cells, at least within the investigated dilution range. Interestingly, the GA4_Pe@DOX emulsion exerted a cytotoxic effect on MCF7 cells very similar to that of the free DOX solution with the same doses of DOX loaded in the same emulsion. In fact, the incorporation of doxorubicin in the ginger powder-based Pickering emulsion did not modify its effect against tumor cells. The drug release from GA4_Pe@DOX was sustained and pH-dependent, with the slowest release at pH 7.4 and the fastest release at pH 5.6. Although further in vivo investigations are already planned to confirm this, the encouraging results on the sustained and pH-dependent release profile allow us to assume that the GA4_Pe platform will be able to provide a new and very safe administration strategy for topical applications of antineoplastic drugs with minimizable adverse side effects.

The lack of in-depth studies on the safety of cutaneous application of DOX-loaded nanoemulsions [36], including those developed for chemo-photodynamic treatment [42,45], suggests the possibility that such formulations may induce adverse side effects—such as edema, pruritus, erythema, and pain—associated with skin reactions at the application site. Such reactions could be attributed to the nature of the photosensitizer, emulsifier, or excipients used. Furthermore, to the best of our knowledge, many DOX-based nanoemulsions may undergo emulsion coalescence and/or chemotherapeutic drug degradation, processes that are not observed in the GA4-Pe@DOX system. Further in vivo studies are certainly needed to verify that topical administration of GA4_Pe@DOX does not cause—or minimizes—any possible side effects associated with the nanoformulation ingredients, all of which are highly biocompatible and naturally rich in antioxidant properties.

## Figures and Tables

**Table 1:** Rheological parameters of Pickering emulsions with different ginger powder concentrations at 25 °C. Different letters for the same parameter refer to significantly different values.

Sample ID	*η_∞_* (Pa s)	*K* (Pa s^n^)	*n* (-)	*A* (Pa s^1/z^)	*z* (-)
05GA4_75Pe	0.0276 ± 0.006	2.2 ± 0.3 ^a^	0.19 ± 0.02 ^a^	92 ± 22 ^a^	6.4 ± 2.0 ^a^
05GA4_100Pe	-	29.9 ± 5.5 ^b^	0.054 ± 0.008 ^b^	587 ± 86 ^b^	7.8 ± 0.7 ^a^
05GA4_200Pe	-	118 ± 14 ^c^	0.065 ± 0.02 ^b^	1680 ± 70 ^c^	7.5 ± 0.8 ^a^

**Table 2:** Rheological parameters of Pickering emulsions with and without doxorubicin. Different letters, for the same parameter, refer to significantly different values.

Sample ID	*η_∞_* (Pa s)	*K* (Pa s^n^)	*n* (-)	*A* (Pa s^1/z^)	*z* (-)
05GA4_75Pe at 37 °C	0.0258 ± 0.004 ^a^	1.02 ± 0.08 ^a^	0.240 ± 0.02 ^a^	32.2 ± 5.6 ^a^	8.9 ± 0.6 ^ab^
05GA4_75Pe@DOX at 37 °C	0.015 ± 0.006 ^b^	0.42 ± 0.05 ^b^	0.427 ± 0.09 ^b^	7.6 ± 1.4 ^b^	14.5 ± 6.7 ^b^
05GA4_75Pe@DOX at 25 °C	0.020 ± 0.005 ^ab^	2.9 ± 0.5 ^c^	0.168 ± 0.03 ^a^	11.6 ± 0.9 ^ab^	5.0 ± 0.2 ^a^

## Data Availability

The data presented in this study are available upon request from the corresponding author.

## Supplementary Materials

The following supporting information can be downloaded at https://www.mdpi.com/article/10.3390/molecules30224349/s1, Figure S1: Images of (A) and (A′) 05GA4_75Pe samples at different φ_w_ values, (B) and (B′) 05GA4_100Pe, and (C) and (C′) 05GA4_200Pe (D1) 1 h and (D2) 1 month after preparation. All samples were stored at 4 °C.; Figure S2. TEM images of multiple droplets of 05GA4_100Pe (scale bar = 100 nm); Figure S3: Droplet size distribution (PSD) of 05GA4_XPe samples freshly prepared () and after 4 weeks of storage at 4 °C (); Figure S4: TEM image of a single droplet in the 05GA4_75Pe sample (scale bar = 100 nm). Table S1: Physical–chemical characterization of GA4 powder in terms of size (nm), PdI, contact angles, and ζ potentials at 25 °C and, expressed as the mean of three independent experiments ± SD; Table S2: The IC_50_ and HillSlope values for DOX, 05GA4_Pe75, and 5GA4_Pe75@DOX for the 3T3 and MCF7 cell viability datasets determined by nonlinear regression analysis using GraphPad Prism 5.0 (GraphPad Software Inc., La Jolla, CA, USA) statistical software.

## Abbreviations

The following abbreviations are used in this manuscript:

GA4	Ginger powder
DOX	Doxorubicin
W/O	Water-in-Oil emulsion
O/W	Oil-in-Water emulsion
W/O/W	Water-in-Oil-in-Water emulsion
BS_TDDS	Biocompatible Sustainable Transdermal Drug Delivery System
FGPS_Pes	Food-Grade Particle-Stabilized Pickering Emulsions
GA4_Pe	Ginger Powder-Stabilized Pickering Emulsion
05GA4_XPe	Ginger Powder-Stabilized Pickering Emulsion with X mg of GA4
GA4_Pe@DOX	Doxorubicin-loaded Ginger Powder-Stabilized Pickering Emulsion
GA4_XPe@DOX	Doxorubicin-loaded Ginger Powder-Stabilized Pickering Emulsion with X mg of GA4
PdI	Polydispersity Index
DLS	Dynamic Light Scattering
POM	Polarized Light Microscopy
SEM	Scanning Electron Microscopy
Cryo-SEM	Cryo-Scanning Electron Microscopy
TEM	Transmission Electron Microscopy
