# Supplementary material for: Ginger Powder-Based Pickering Emulsions: An Innovative Platform for Anticancer Drug Delivery
Source: Molecules. 2025 Nov 10;30(22):4349. doi: 10.3390/molecules30224349 (PMC12655056; doi:10.3390/molecules30224349)
Supplement: Supplementary file 1 [file molecules-30-04349-s001.zip › molecules-3795966-supplementary.pdf]

Supporting Information

Table S1. Physical-chemical characterization of GA4 powder in terms of size (nm), PdI, contact angles, and ζ potentials, at 25°C, and expressed as the mean of three independent experiments ± SD.

	**Size (nm)**	**PdI**	**Contact angle (°)**		**ζ potential mV (pH 5.6)**	**ζ potential mV (pH 7.4)**
			**Water**	**Mygloil®812N**		
GA4	370.2 ± 20.6	0.15 ± 0.07	101 ± 3	36 ± 4	−12.77 ± 0.58	−14.30 ± 0.43

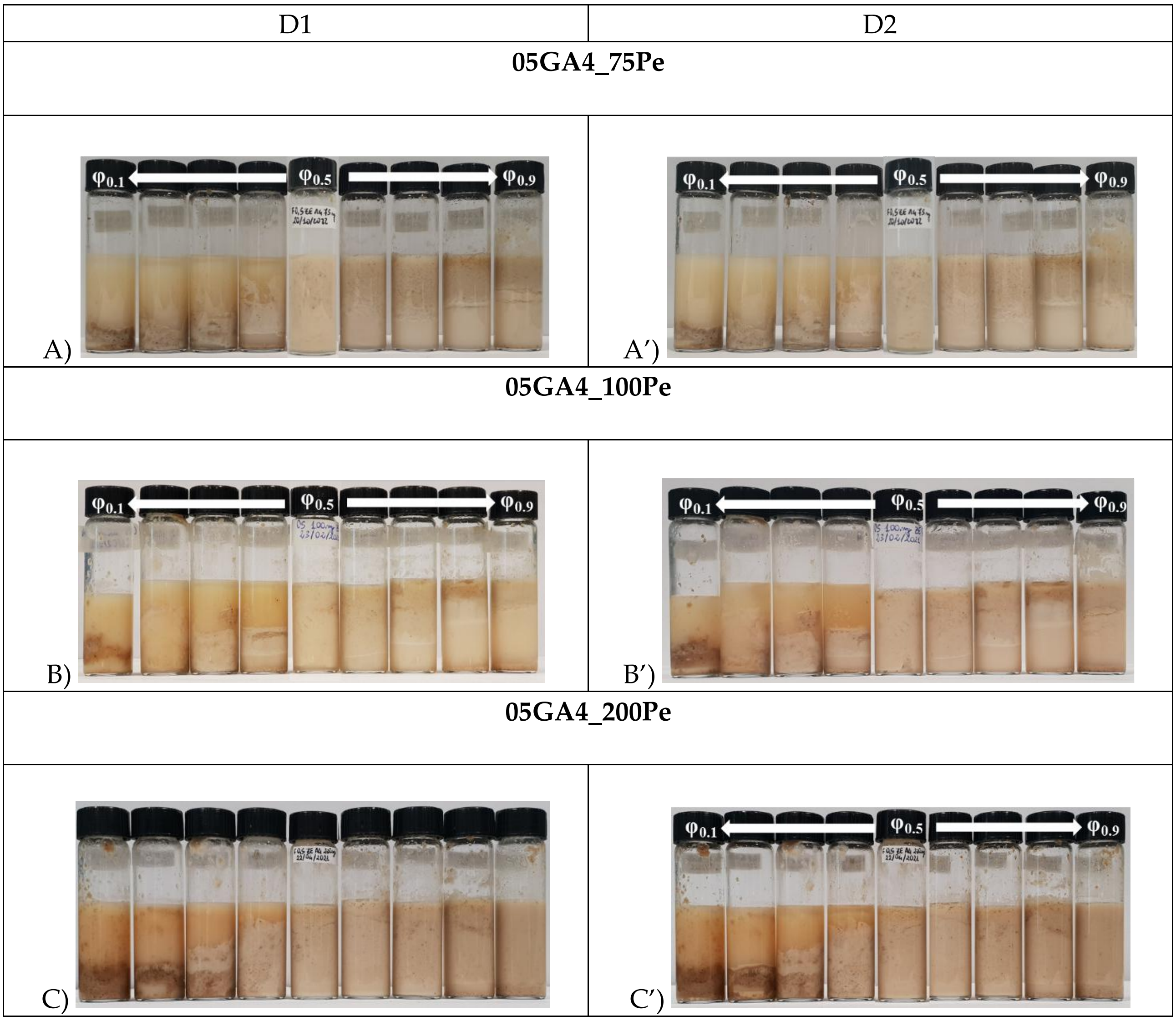

Figure S1. Images of A) and A') 05GA4_75Pe samples at different φ_w*values, B) and B') 05GA4_100Pe, and C) and C') 05GA4_200Pe after (D1) 1h and (D2) 1 month after preparation. All samples were stored at 4°C.

$$* \varphi_w = V_{water} / (V_{water} + V_{Miglyol^{®}812N}) \qquad \textbf{Eq.S1}$$

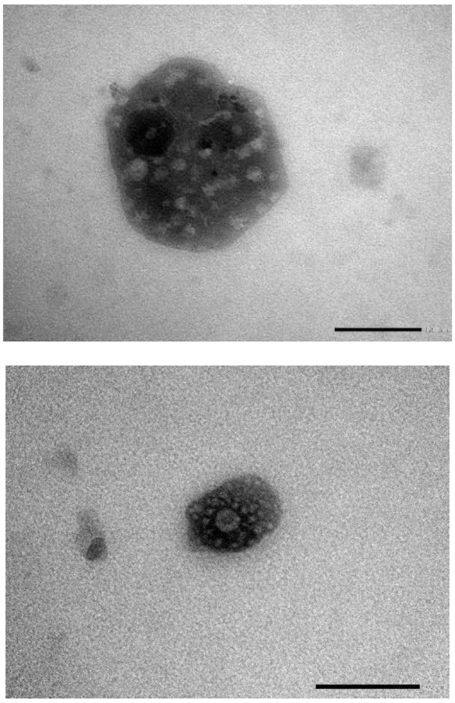

Figure S2. TEM images of multiple droplets of 05GA4_100Pe (scale bar = 100 nm).

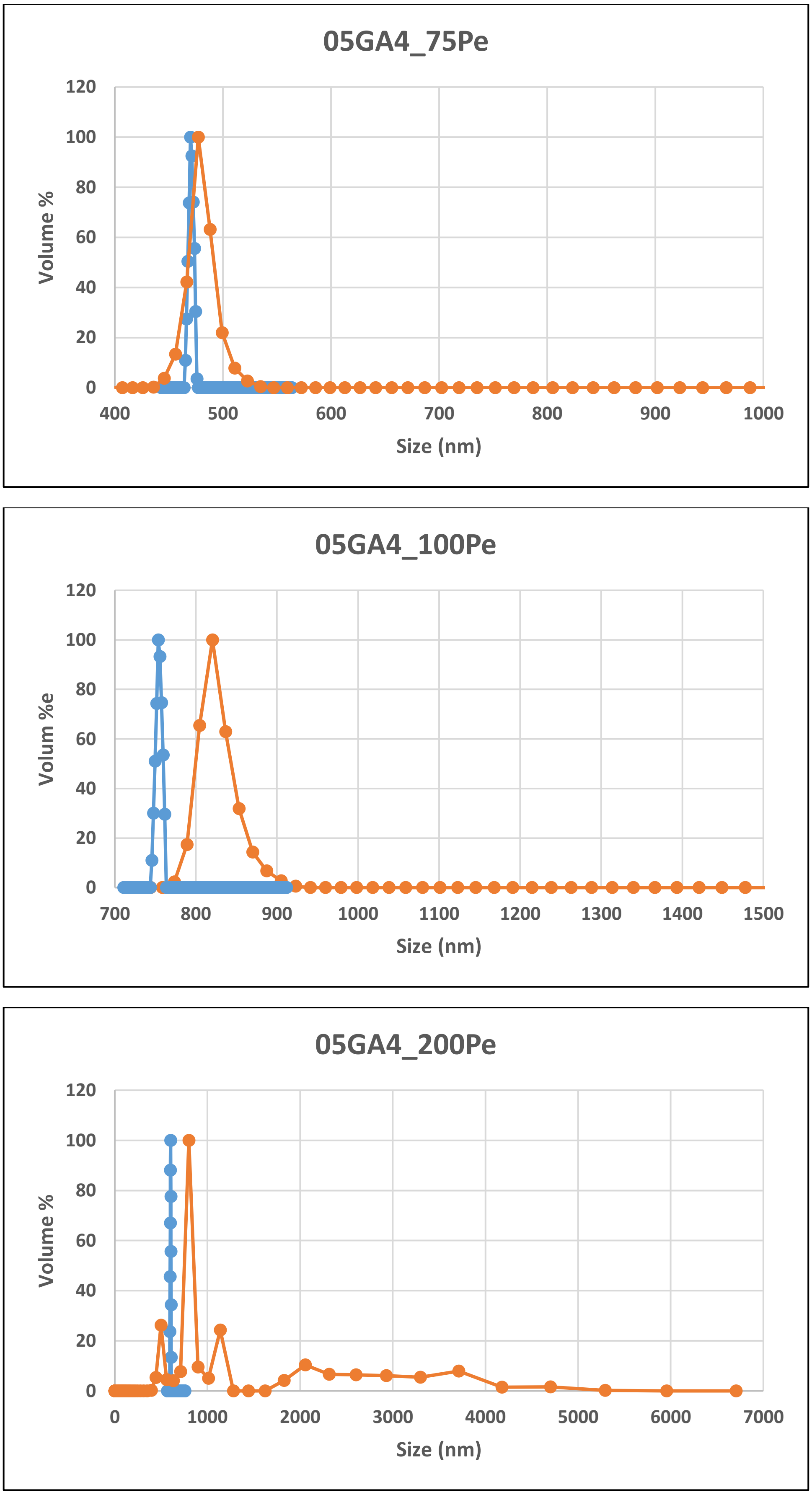

Figure S3. Droplet Size Distribution (PSD) of 05GA4_XPe samples freshly prepared (—●—) and after 4 weeks of storage at 4° C (—●—).

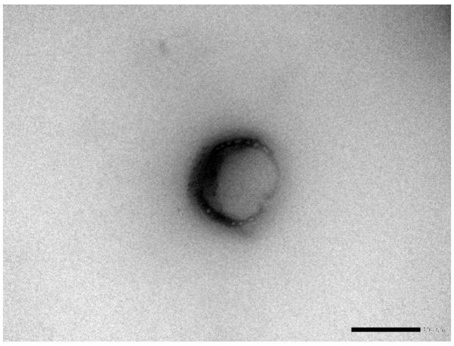

Figure S4. TEM image of a single droplet in the 05GA4_75Pe sample (scale bar = 100 nm).

Table S2. The IC_{50} and HillSlope values for DOX, 05GA4_Pe75, and 5GA4_Pe75@DOX for the 3T3 and MCF7 cell viability datasets determined by nonlinear regression analysis using GraphPad Prism 5.0 statistical software.

	**Cell line**	**IC_{50} (µg/ml)***	**Hill Slope**
05GA4_Pe75	3T3	Not detectable	−0.00352
DOX	MCF7	0.3570<br>(0.2937–0.4339)	−0.3808
05GA4_75Pe@DOX	MCF7	0.3623<br>(0.2943–0.4462)	−0.4837
05GA4_Pe75	MCF7	Not detectable	−0.03091

*Numbers in parentheses are 95% confidential intervals.
